# Immunomodulatory Properties of *Streptococcus* and *Veillonella* Isolates from the Human Small Intestine Microbiota

**DOI:** 10.1371/journal.pone.0114277

**Published:** 2014-12-05

**Authors:** Bartholomeus van den Bogert, Marjolein Meijerink, Erwin G. Zoetendal, Jerry M. Wells, Michiel Kleerebezem

**Affiliations:** 1 Top Institute Food and Nutrition (TIFN), Wageningen, The Netherlands; 2 Laboratory of Microbiology, Wageningen University, Wageningen, The Netherlands; 3 Host-Microbe Interactomics Group, Wageningen University, Wageningen, The Netherlands; 4 NIZO Food Research B.V., Ede, The Netherlands; Institut Pasteur de Lille, France

## Abstract

The human small intestine is a key site for interactions between the intestinal microbiota and the mucosal immune system. Here we investigated the immunomodulatory properties of representative species of commonly dominant small-intestinal microbial communities, including six streptococcal strains (four *Streptococcus salivarius*, one *S. equinus*, one *S. parasanguinis*) one *Veillonella parvula* strain, one *Enterococcus gallinarum* strain, and *Lactobacillus plantarum* WCFS1 as a bench mark strain on human monocyte-derived dendritic cells. The different streptococci induced varying levels of the cytokines IL-8, TNF-α, and IL-12p70, while the *V. parvula* strain showed a strong capacity to induce IL-6. *E. gallinarum* strain was a potent inducer of cytokines and TLR2/6 signalling. As *Streptococcus* and *Veillonella* can potentially interact metabolically and frequently co-occur in ecosystems, immunomodulation by pair-wise combinations of strains were also tested for their combined immunomodulatory properties. Strain combinations induced cytokine responses in dendritic cells that differed from what might be expected on the basis of the results obtained with the individual strains. A combination of (some) streptococci with *Veillonella* appeared to negate IL-12p70 production, while augmenting IL-8, IL-6, IL-10, and TNF-α responses. This suggests that immunomodulation data obtained *in vitro* with individual strains are unlikely to adequately represent immune responses to mixtures of gut microbiota communities *in vivo*. Nevertheless, analysing the immune responses of strains representing the dominant species in the intestine may help to identify immunomodulatory mechanisms that influence immune homeostasis.

## Introduction

The human intestine is home to a myriad of different microbial organisms, most of which are bacteria [1] and collectively known as microbiota. The intestinal microbiota is of particular interest because it plays an essential role in the maturation and development of the mucosal immune system in early life [2], [3] and the preferential tolerance induction to harmless antigens at mucosal sites [4], [5]. The contribution of individual microbes to the mechanisms that maintain immune homeostasis are just beginning to be understood [1], [6], [7], but their importance is highlighted by the disturbances in microbiota composition associated with several intestinal-related diseases including obesity, multiple sclerosis, inflammatory bowel diseases, and type 1 diabetes [3], [8], [9], [10], [11], [12], [13]. Research on this topic has been biased towards the analysis of fecal samples that only provide information about the microbiota at the end of gastrointestinal (GI) tract [13], [14], [15], [16], meaning that, the immune-influences driven by microbial communities in the upper intestinal tract have been largely neglected [17]. This is mainly attributable to the limited accessibility of the small intestine. Nevertheless, the Peyer's patches (PP) of the small intestine are major sites for sampling of luminal antigens, including bacteria, and the induction of adaptive immune responses. Antigen sampling by the follicle–associated epithelium (FAE) overlaying the lymphoid follicles of the PP is facilitated by the lack of mucin secreting goblet cells and the presence of specialized Microfold cells (M cells) [2], [18], [19]. Bacteria sampled by M cells in the FAE are transported intact to the sub-epithelial dome of PP where dendritic cells (DCs) play a key role in bacterial handling and the induction of subsequent immune responses (see [20] for a review). Recently, PP dendritic cells were shown to sample bacteria and antigens through M cell-specific transcellular pores [21], [22]. Additionally, CX3CR1+ cells in the epithelium expressing DC or macrophage markers have been shown to sample luminal bacteria (and other luminal constituents) directly in the lumen by passing protrusions through the paracellular space of the epithelium without disrupting epithelial integrity [23], [24], [25].

While both the human small and large intestinal microbiota encompasses anaerobes belonging to the *Clostridium* clusters, the marked difference between these intestinal niches is a microbial composition predominated by facultative anaerobes, including the streptococci and *Veillonella* bacteria in the small intestine [17], [26], [27] (Leimena and Van den Bogert, et al., Unpublished data). The co-occurrence of these genera may in part depend on their potential for metabolic interaction as shown in the oral cavity [28] and previously postulated for the small intestine [17]. Support for this notion comes from the high expression of genes involved in primary carbohydrate transport systems by the small intestinal streptococci [17], indicating a role for the *Streptococcus* populations as primary fermenters of diet-derived simple sugars in the human small intestine. Characterization of small-intestinal bacterial streptococci revealed that the small intestine is inhabited by a variety of *Streptococcus* lineages that belong to *S. parasanguinis*, *S. equinus*, and *S. salivarius* species. These lineages displayed considerable phenotype variability in terms of carbohydrate utilization capacities [29], [30], which was in excellent agreement with their capacities predicted on basis of their genome sequences [30]. With the exception of streptococci, the lactic acid bacteria are generally present at low abundance in the small intestine microbiota [26], [30], [31] (Leimena and Van den Bogert, et al., Unpublished data), but nevertheless display a substantial level of phylogenetic richness in individuals, as was also concluded for members of the genus *Enterococcus* [29]. The enterococci are common colonizers of the GI tract, but have a less attractive reputation because of the pathogenic potential of specific members of this genus [32].

Considering the prominent role of DCs in modulation of the small-intestinal immune system the aim of the current study was to investigate the immunomodulatory properties of different small-intestinal *Enterococcus*, *Streptococcus*, and *Veillonella* isolates [29], with a special focus on the latter two genera because of their predominance in the small-intestinal ecosystem.

## Materials and Methods

### Bacterial strains

Six *Streptococcus* strains (with known genome sequences; [29], [30]), an *Enterococcus gallinarum* HSIEG1 strain [33], and a *Veillonella parvula* HSIVP1 strain [29], [34], as well as the reference strain *Lactobacillus plantarum* WCFS1 [35] were used in the immunoassays (Table 1). The streptococcal strains were representative isolates of 6 distinct phylogenetic lineages, as determined by DNA fingerprinting, belonging to: *S. parasanguinis* (1 strain; HSISM1), *S. equinus* (1 strain; HSISB1), and *S. salivarius* (4 strains; HSISS1-4; Table 1) [29], [30]. The streptococcal and *Enterococcus* strains were grown in *Mitis*-*Salivarius* (MS) medium [29], while *Veillonella* was grown in medium described in the DSMZ catalogue (Medium 136) under anoxic N_2_ atmosphere. *Lactobacillus plantarum* WCFS1 was grown in Mann-Rogosa Sharpe (MRS) medium (Becton Dickinson, Breda, The Netherlands). Fresh culture media did not induce any cytokine responses (data not shown). All strains were twice subcultured overnight successively, after which the streptococci and the *Enterococcus* strains had an average OD_600_ of 1.3 (± standard deviation of 0.2), while the *V. parvula* strain and WCFS1 had OD600 of approximately 0.5 and 2.5, respectively. The bacteria suspsensions were diluted in PBS (GIBCO) to a final OD_600_ of 1.

**Table 1:** Strains used in this study.

Species	Strain identifier
*Streptococcus parasanguinis*	HSISM1*
*Streptococcus equinus*	HSISB1*
*Streptococcus salivarius*	HSISS1*
*Streptococcus salivarius*	HSISS2*
*Streptococcus salivarius*	HSISS3*
*Streptococcus salivarius*	HSISS4*
*Veillonella parvula*	HSIVP1*
*Enterococcus gallinarum*	HSIEG1*
*Lactobacillus plantarum*	WCFS1**

*: Strain was cultivated from ileostoma effluent [29], [30].

**: Strain was cultivated from human saliva [35].

### Differentiation and maturation of dendritic cells

The study was approved by the Wageningen University Ethical Committee and was performed according to the principles of the Declaration of Helsinki. Buffy coats were obtained from the Sanquin Blood bank Nijmegen, the Netherlands. A written informed consent was obtained before sample collection. Human monocytes were isolated from blood using a combination of Ficoll density centrifugation and cell separation using CD14-specific antibody coated magnetic microbeads (Miltenyi Biotec, Leiden, the Netherlands). The purity of isolated CD14+ cell fraction was greater than 90% and cell-viability was above 95% in all experiments. To generate immature DC (iDCs), the purified CD14+ cells were cultured for 6 days in RPMI 1640 medium (Invitrogen, Breda, the Netherlands), supplemented with 100 units/ml penicillin G (Invitrogen), 100 µg/ml streptomycin (Invitrogen), 50 ng/ml IL-4 (R&D systems, Abingdon, United Kingdom) and 50 ng/ml granulocyte-macrophage colony-stimulating-factor (GM-CSF) (R&D systems). GM-CSF and IL-4 were added to differentiate the monocytes into myeloid DCs. On day 6 approximately 1×10^6^ iDCs were stimulated with LPS (1 µg/ml) or the different bacteria at a cell to bacteria ratio of approximately 1∶1 and 1∶10 for 24 hours. As anticipated and as a consequence of the supplementation of the cell-media with antibiotics, no bacterial growth was observed during this period. Non-stimulated iDCs were used as a negative control.

### Analyses of cell surface markers and measurement of cell death by flow cytometry

On days 3, 6, and 8 the percentage of viable cells was measured by flow cytometry (FACSCanto II, BD, San Diego, USA). Live, apoptotic and necrotic cells were discriminated by staining with Annexin V and propidium iodide (PI) according to the manufacturer's protocol. The cells were analysed using flow cytometry (FACSCanto II, BD, San Diego, USA) and the BD FACSDiva software. Cells that are negative for both Annexin V and PI are not apoptotic or necrotic as translocation of the membrane phospholipid phosphatidylserine has not occurred and the plasma membrane is still intact. Therefore, Annexin V and PI double negative cells were considered as viable cells, whereas both single and double positive cells were regarded as non-viable [36]. On days 3 to 8 the viability of the cells was between 60 and 95%. There were no significant differences in cell death between the bacteria-stimulated cells and the non-stimulated (negative control) or LPS-stimulated (positive control) cells.

On day 8, cells were also stained with fluorescence-conjugated monoclonal antibodies specific for CD83, CD86 or their isotype-matched controls (BD biosciences, San Diego, USA) and analysed by flow cytometry (FACSCanto II, BD, San Diego, USA) to check the maturation and activation status of the cells. CD83 and CD86 are highly expressed on DCs after stimulation with known maturation factors (e.g. LPS) compared to non-stimulated immature dendritic cells. The expression of CD83 and CD86 from different human donors can vary considerably after stimulation with different stimuli so for comparison the data was normalized to the values (100%) obtained using a standard amount of LPS added to cells from each donor.

### Cytokine assays

Supernatants from the DC stimulation assays were collected after stimulation for 24 hours, and analysed for the presence of cytokines (IL-1β, IL-6, IL-8, IL-10, IL-12p70 and TNF-α) using a cytometric bead-based BD Human inflammation kit that enables multiplex measurements of soluble cytokines in the same sample [37], according to the manufacturer's protocol (BD biosciences, Breda, the Netherlands). The sensitivity-limits of detection were as follows: IL-1β 7.2 pg/ml, IL-6 2.5 pg/ml, IL-8 3.6 pg/ml, IL-10 3.3 pg/ml, IL-12p70 1.9 pg/ml and TNF-α 0.7 pg/ml. The flow cytometry data were analysed using the BD FCAP software (Figure S1). Unless stated otherwise, cytokine secretion in the remainder of the paper are based on stimulation of iDCs with a DC to bacteria ratio of approximately 1∶10.

### Disruption of bacterial cells and spent medium collection

To determine whether the *S. equinus* strain might possess an immunomodulatory component, which suppresses cytokine secretion, the *S. equinus* as well as *S. salivarius* strain 4 were disrupted using a cell disruptor (LaBiosystems, Constant systems, Waalwijk, The Netherlands). The latter strain was chosen as a control strain because of its capacity to induce considerable cytokine production levels in dendritic cells. In addition spent medium was collected, by centrifugation of an overnight culture of *S. equinus* or *S. salivarius* strain 4.

### TLR2/6 assay

TLR2/6 signalling capacities of the bacterial strains were determined using a reporter assay with Human Embryonic Kidney (HEK) 293 (Invivogen, Toulouse, France) cells expressing human TLR2 and TLR6 heterodimers that recognize lipoteichoic acid (LTA) and lipoprotein lipid anchors of Gram-positive bacteria [38]. The TLR2/6 signalling assay was performed essentially as previously described [39]. Briefly, HEK293 cells were transformed with human TLR2/6 and pNIFTY, a NF-κB luciferase reporter construct (Invivogen, Toulouse, France). HEK293 cells transformed with only the pNIFTY did not respond to Pam_2_CSK (20 ng/ml) (synthetic agonist of TLR2/6) demonstrating the dependency of NF-κB activation on co-expression of hTLR2/6 receptor [39]. The cells were plated at a concentration of 6×10^4^ cells per well in DMEM medium (Invitrogen). Cells were then stimulated with the different bacterial strains, or Pam2CSK (20 ng/ml) as a positive control or with medium alone (negative control) followed by incubation at 37°C for 6 hours under a 5% CO_2_ atmosphere. Thereafter, the medium was replaced with Bright glow (Promega, Leiden, the Netherlands), and the plates shaken for 5 minutes before measuring the luminescence in a Spectramax M5 (Molecular Devices, Sunnyvale, United States). HEK293 cells not expressing TLR receptors that harbour pNIFTY were used as the negative control in the NF-κB assays.

### Statistical analysis

Mixed general linear model using restricted maximum likelihood (REML) was used to determine the statistical differences within donors between cytokine produced by DCs stimulated with the different bacterial strains. A two-sided *p*-value of 0.05 or lower was considered to be significant. The statistical analysis for the cytokine secretion by dendritic cells after mono-stimulation with bacterial strains, disrupted strains, and spent medium a One-Way ANOVA test was used to compare the cytokine secretion between bacteria stimulated cells, as the group size (n = 2) was not sufficient using REML. The statistical analysis (REML) was performed by using SAS software (version 9.1, SAS Institute Inc., Cary, NC, USA) and the One-Way ANOVA test Graphpad Prism5.

## Results

### Small-intestinal bacteria differentially affect DC maturation and activation

**Figure 1:**
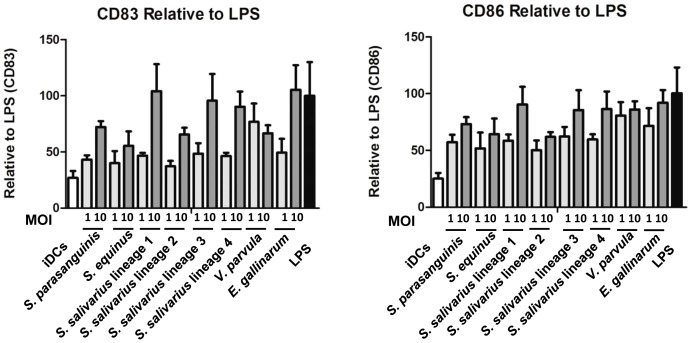
MFI of stained cell surface markers CD83 (A) and CD86 (B) by monocyte derived dendritic cells. Immature DCs were used as the negative control and LPS as the positive control. Dendritic cells were derived from monocytes of 5 different human donors.

*S. parasanguinis* HSISM1, *S. equinus* HSISB1, 4 different *S. salivarius* strains (HSISS1-4), *E. gallinarum* HSIEG1, and *V. parvula* HSIVP1 strains obtained from the human small intestine were investigated for their capacity to induce maturation and activation of immature monocyte-derived DCs from donors. The DCs were stimulated for 24 hours with different strains at DC to bacteria ratios of 1 and 10. The expression of the surface marker CD83 (maturation marker) and CD86 (maturation marker and co-stimulatory molecule) were measured to determine maturation and activation status of the DCs. The mean fluorescence intensity (MFI) of dendritic cells was normalized to LPS stimulation (Figure 1). Stimulation of the DCs by all strains with the high dose (1 to 10) resulted in higher maturation and activation marker expression compared to the medium control, except for CD83 induced by *S. equinus* (Table S1). Furthermore, significant differences (p<0.05) were observed between the different ratios for *S. parasanguinis*, and *S. salivarius* 1, 2, and 4. The induction of the expression of the surface markers CD83 differed markedly among the different species used as DC stimulants. *S. salivarius* strain 1, 3, and 4 and *E. gallinarum* induced highest expression (Figure 1). Moreover, while *V. parvula* induced moderate levels of CD83 expression, whereas it was one of the strongest inducers CD86 expression among the tested strains.

### DC cytokine responses to bacterial isolates from the small intestine

The small-intestinal *Streptococcus*, *Veillonella* and *Enterococcus* strains were further investigated for their capacity to induce cytokine secretion by monocyte-derived iDCs. In addition, *L. plantarum* WCFS1 was employed as a benchmark strain that was analysed several times before [40], [41], [42], [43]. The IL-10 and TNF-α levels induced by *L. plantarum* WCFS1 were comparable to a previous study [41]. Noteworthy, *L. plantarum* WCFS1 induced considerably higher amounts of IL-8, IL-6, and IL-10, higher than the streptococci, albeit that this was based on DCs derived from 2 donors (Table S2).

**Figure 2:**
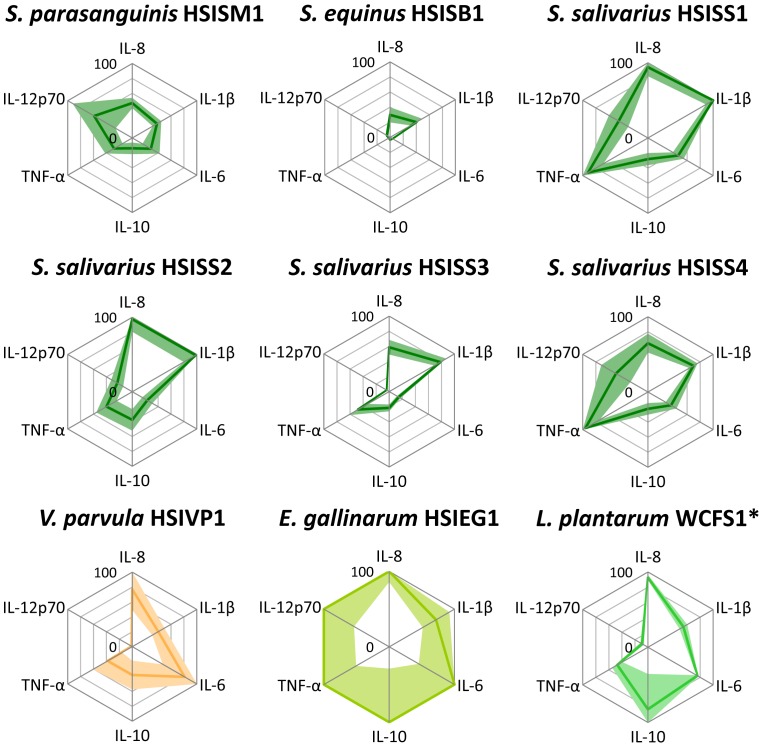
Cytokine secretion by dendritic cells after stimulation with bacterial strains. Dendritic cells were derived from monocytes of 5 human different donors. Cytokine levels are expressed as relative values of the highest inducing strain (100% cytokine levels (pg/ml): IL-8: 17598; IL-1β: 41; IL-6: 4775; IL-10: 206; TNF-α: 5151; IL-12p70: 2397; Table S2). Lines represents the average secreted cytokine amounts and faded colours represent the interval between the upper and the lower SEM. SEM values higher than 100% are not visualized. *: Cytokine responses determined using DCs derived from 2 different human donors (Figure 5, Table S2).

Although cytokine responses upon stimulation with the different bacterial strains varied between the different donors, the induced immune profiles were consistent (Figure 2). The *V. parvula* strain elicited a moderate induction of the production of the cytokines IL-8, IL-1β, IL-10, and TNF-α. In contrast to the *Streptococcus* strains, *V. parvula* stimulated hardly any IL-12p70 secretion in DCs, whereas its capacity to induce IL-6 was substantially higher, albeit that this was not significant (Figure 2; Table S2, S3, S4, and S5).

Although, the cytokine response between donors for the *E. gallinarum* strain varied, the averaged induced cytokine amounts by *E. gallinarum* strain consistently were the highest among the tested strains (Figure 2; Table S2), indicating that the immune system response is more pronounced if triggered with this strain compared to the streptococci.

Although the IL-1β (16–41 pg/ml) and IL-10 (7–78 pg/ml) levels induced by the streptococci varied (Table S2), the levels were relatively low. In agreement with what has previously been described for members of the *S. bovis* species group [44], the *S. equinus* strain tested here consistently induced low levels of cytokines (Figure 2). This was especially clear for the significantly lower IL-8, IL-6, IL-10, and TNF-α levels induced by *S. equinus* compared to those by the *S. salivarius* strains (p<0.05; Table S2, S3, S4, and S5).

The *Streptococcus* strains showed substantial differences in their ability to induce the production of the chemokine IL-8 (5231–17147 pg/ml) and the pro-inflammatory cytokines IL-6 (161–2221 pg/ml), TNF-α (86–4933 pg/ml), and IL-12p70 (81–1416 pg/ml; Figure 2; Table S2, S3, S4, and S5). This illustrates that the strains tested here elicited distinct cytokine profiles, which is in agreement with earlier observations that revealed distinct DC responses to closely related species and strains [43], [45]. The immune response profiles elicited by the *S. salivarius* strains 1 and 4 were not significantly different (Table S3, S4, and S5), except for IL-1β that was induced at a low (but significantly different) level by both strains (see also above). This corroborates earlier observations on the close relatedness of these two *S. salivarius* strains that were based on genetic fingerprinting and physiological evaluations [29], [30].

### *S. equinus* is not immunosuppressive

**Figure 3:**
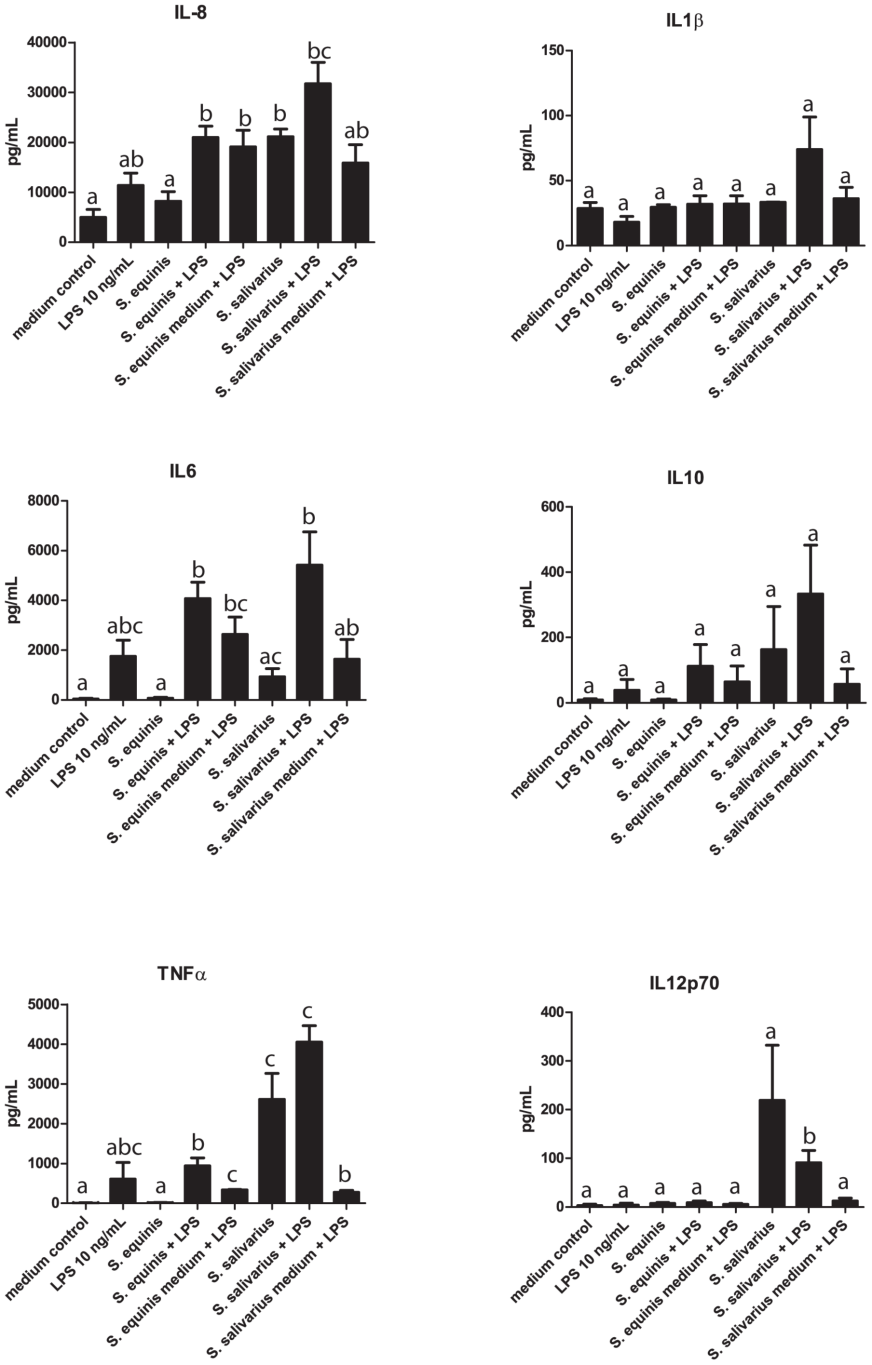
Cytokine secretion by dendritic cells after mono-stimulation with bacterial strains, disrupted strains, and spent medium. Dendritic cells were derived from monocytes from 2 human donors. Spent medium was tested with or without LPS.

As the *S. equinus* strain elicited a low immune response compared to the other strains tested (Figure 2), we hypothesized that this strain might possess an immunomodulatory component that suppresses cytokine secretion. Therefore, we co-stimulated DCs with LPS (10 ng/ml) and *S. equinus* or *S. salivarius* strain 4. A lower LPS dose was used (10 ng/mL) compared to the previous immune assay (mono-stimulations) to be able to modulate the cytokines response. *S. salivarius* strain 4 was chosen as a control strain because it induced considerable cytokine production in dendritic cells. Moreover, the genomic lineage that this strain belongs to was highly predominant in ileostoma effluent and appears to be among the genomic lineage that is ubiquitously found in the human small intestine, supporting the relevance of selecting this strain for comparative reasons [29], [30]. The *S. equinus* strain did not significantly modulate the cytokine levels induced by LPS stimulation. Nevertheless, the amount of IL-6 produced by DCs stimulated with *S. equinus* and LPS together, was higher than the sum of the levels induced by the two separate stimuli, suggesting that these stimuli may synergistically induce the secretion of this cytokine by DCs rather than the hypothesized immunosuppressive effect of *S. equinus*. A qualitatively similar and quantitatively significant synergistic effect on IL-6 production was also observed when DCs were co-stimulated with *S. salivarius* and LPS (Figure 3). Co-stimulation of DCs with LPS and spent culture supernatant from either of the two bacterial strains also consistently elevated production of most cytokines (except for TNF-α) as compared to LPS alone (Figure 3), although this effect was not significant and appeared to be smaller as compared to the co-stimulation by the bacterial cells.

Taken together these results establish that *S. equinus* displays no detectable immunosuppressive effect on dendritic cell cytokine production levels, but appears to be able to moderately enhance cytokine production in response to LPS.

### Involvement of TLR2 and TLR6 in innate immune signalling by small-intestinal *Streptococcus* and *Veillonella* strains

**Figure 4:**
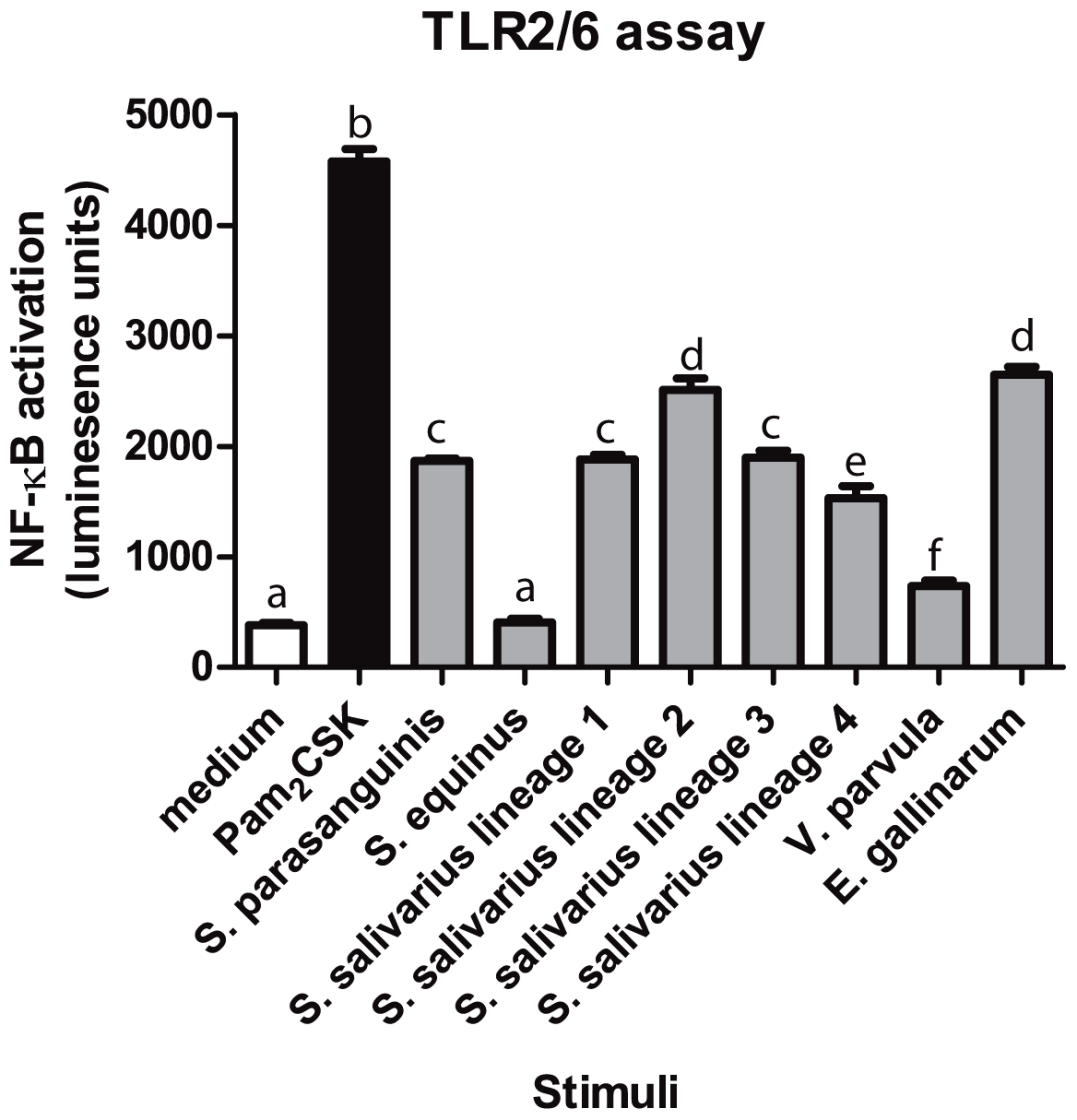
TLR2/6 signalling capacities of bacterial strains. HEK293 cells were incubated with the small-intestinal strains at a cell to bacteria ratio of 1∶10, PAM_2_CSK as a positive control and medium as a negative control (n = 3). This figure is representative out of two hTLR2/6 assays.

TLR2/6- mediated activation of NF-κB is potentially one of the major pathways for DC activation via LTA or lipoproteins derived from the cell envelope of bacteria. Therefore, we tested the TLR2/6 signalling capacities of the *Streptococcus* and *Veillonella* strains in a reporter assay using HEK293 cells expressing human TLR2 and TLR6 heterodimers that recognize lipoteichoic acid (LTA) and lipoprotein lipid anchors of Gram-positive bacteria [38]. The results demonstrated that most strains are capable of triggering NF-κB activation via TLR2/6 dependent signalling. A notable exception is the *S. equinus* strain that did not significantly induce TLR2/6 signalling in this reporter assay (Figure 4), which is analogous to its failure to induce high levels of cytokine production in DCs (see above). Similarly, the strong DC-response elicited by *E. gallinarum* stimulation was reflected in its TLR2/6 signalling capacity, where this strain classified as one of the strongest TLR2/6 stimulators among the strains tested. These results suggest a certain degree of congruency between the capacity of individual strains to elicit TLR2/6 signalling in HEK293 NF-κB reporter cells, and their capacity to stimulate high levels of cytokine production in iDCs.

### Co-stimulation of dendritic cells with streptococci and *Veillonella*

Based on the frequent co-occurrence of the *Streptococcus* and *Veillonella* spp. in various habitats associated with the human body [17], [28], we evaluated the cytokine responses that were elicited in iDCs that were co-stimulated with one of the small intestinal *Streptococcus* strains in combination with the *V. parvula* strain.

**Figure 5:**
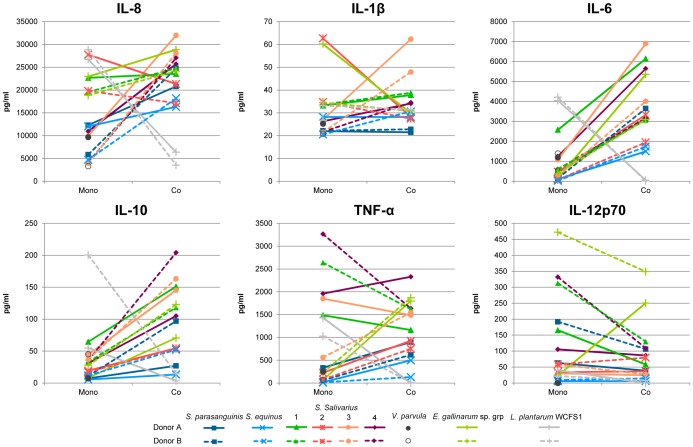
Cytokine secretion by dendritic cells after mono-stimulation with tested strains and co-stimulation with *V. parvula*. Dendritic cells were derived from 2 human donors. Mono-stimulations (Mono)_ and co-stimulations (Co) were both performed at a cell to bacteria, or a cell to a combination of two strains, ratio of approximately 1∶10. See table S6 for cytokine values.

Though cytokine production levels differed for DCs derived from different donors, the overall response profiles induced by a combination of the two species differed from the levels anticipated on basis of mono-stimulation with the individual strains (Figure 5).

As an example of the specific co-stimulatory effects, the amounts of TNF-α secreted upon co-stimulation of iDCs with *V. parvula* and the *S. parasanguinis*, *S. equinus*, and *S. salivarius* strain 2 were considerably increased in comparison to those observed with mono-stimulations with either one of the streptococci or the *V. parvula* strain alone. Notably, this effect was not observed for co-stimulation of *V. parvula* together with *S. salivarius* strain 1, which actually led to decreased TNF-α production as compared to the cognate mono-stimulations. The DC response to co-stimulation with *V. parvula* and *S. salivarius* strain 3 and 4, appeared to vary between multiple donors. Finally, while mono-stimulation with the *Streptococcus* strains induced variable amounts of IL-8 and generally low amounts of IL-6 and IL-10 production in DCs, co-stimulation of DCs commonly led to higher amounts of secreted IL-8, IL-10, and IL-6 (Figure 5; Table S6). The latter observation was especially obvious for co-stimulation with *V. parvula* and the *S. equinus* or *S. salivarius* strains 1, 3, and 4, which by themselves induced among the lowest amounts of IL-8 and IL-6 of all tested bacteria (Table S6), but in combination with *V. parvula* induced high amounts of these cytokines in iDCs (Figure 5; Table S6).

Interestingly, the postulated synergy between *V. parvula* and the streptococci with respect to stimulation of production of IL-8, IL-6, IL-10, and TNF-α might be relatively specific for these combinations of bacteria, as co-stimulation of iDCs with *V. parvula* and *L. plantarum* WCFS1 suppressed production of these cytokines, leading to the lowest IL-8, IL-6, IL-10, and TNF-α amounts observed in these co-stimulation analyses (Figure 5; Table S6). These observations suggest that immune cell stimulation with combinations of some streptococci and *V. parvula* may elicit responses that are specific for the combined bacterial stimuli, leading to immune-synergistic effects that could not be predicted from respective mono-stimulations with either of the bacteria.

Extrapolation of these *in vitro* immune (co-)stimulation profiles to the *in vivo* situation that encompasses the exposure of the immune system to bacterial communities rather than single strains is far from trivial. Nonetheless, certain trends could be seen in our *in vitro* results of streptococcal and *V. parvula* co-stimulation, suggesting at least a partial consistency in the co-stimulatory capacities of two species. This notion is further illustrated by the high similarity of the immune profiles elicited by co-stimulation with *V. parvula* and *S. salivarius* strain 1 or 4 (Figure 5), which is in good agreement with the close relatedness of these streptococcal strains ([29], [30]; see also above).

## Discussion

Individual GI commensals (e.g. *Faecalibacterium prauznitsii* [46] and *Bacteroides fragilis* [47], [48]) affect the host immune system in specific ways (see [49] for a recent review). Given that the human small intestine is an important region to study host-microbe interactions, we evaluated the immunomodulatory properties of *Streptococcus, Veillonella*, and *Enterococcus* strains isolated from the small intestine. The strains used (especially valid for the streptococci [29]) can be regarded as representatives of distinct phylogenetic lineages that were identified among a large panel of isolates obtained from the human small intestine ecosystem. The *Streptococcus* strains tested here, have previously been subjected to in depth analysis, including physiological studies focussing on their carbohydrate utilizing capacities [29] and the determination of their complete genome sequences [30]. The current study revealed that these *Streptococcus* strains differ significantly in their ability to elicit cytokine production responses in iDCs as well as their capacity to activate NF-κB responses via TLR2/6. These findings are in agreement with previous reports that conclude that significantly different immunomodulatory properties can be observed in the comparison of closely related species [45] and strains [43]. However, stimulation of iDCs with *S. salivarius* strain 1 and 4 induced similar amounts of different cytokines, which is in agreement with their highly conserved genetic content and physiological characteristics [29], [30]. Among the strains tested, *S. salivarius* strain 2 was the least effective in activating and maturating responses in iDCs, but at the same time was identified as one of the strongest inducers of TLR2/6 signalling, which is likely due to the difference in phagocytosis capacity between dendritic cells and HEK293 cells or that bacterial components are shielding certain microbe-associated molecular patterns (MAMPs) The cytokine responses of the small intestinal streptococci were quite similar to other *Streptococcus* strains, including pathogenic *S. suis* strains although these elicited higher IL-12 (up to 6948 pg/ml) in DCs [50]. However, the small intestinal *Streptococcus* strains tested here are not known to be virulent, although remnants of streptococcal virulence genes were identified in their genomes [30]. Similarly, remnants of virulence related genes were also encountered in the genomes of strains of the yoghurt-associated species *S. thermophilus* [51], suggesting that benign streptococci may share functions with related pathogens.

Compared to the streptococci, the small intestinal *E. gallinarum* strain appeared to be consistently more potent in inducing cytokine production in iDC and was one of the strongest inducers of TLR2/6 signalling, which is in agreement with earlier studies that report on the highly immune-stimulating capacities of enterococci [52], [53].

In contrast to the other streptococci tested in this study, DCs were relatively unresponsive to the *S. equinus* strain, which also induced negligible TLR2/6-mediated signalling. Interestingly, the amounts of cytokines produced by DCs co-stimulated with *S. equinus* and LPS were higher compared to stimulation with LPS alone, indicating a synergistic immunostimulatory effect. The low immune response to *S. equinus* may therefore be due to the modification of conserved MAMPs reducing their capacity to signal through TLRs and NLRs or shielding effects (e.g. due to capsule polysaccharide). Close relatives of the *S. equinus* strain (e.g. *S. gallolyticus* subsp *gallolyticus* UCN34 [54]) have a less attractive reputation and are known to evade the host immune system and have been associated with GI tract malignancies [44]. Notably, genome mining of the *S. equinus* strain [30] revealed gene repertoires similar to the capsular operon encoded by *S. gallolyticus* subsp *gallolyticus* UCN34 [54] (data not shown), which was postulated to shield the bacterial cell from the host immune system [44]. Further comparative analyses could elucidate the genetic relatedness (e.g. coding capacities for virulence factors) between the *S. equinus* strain tested here and potentially pathogenic close relatives.

As *Streptococcus* and *Veillonella* spp. have been found to co-occur in various microbial ecosystems associated with humans and are proposed to have metabolic interactions [17], [28], the small-intestinal isolates from both genera were tested in co-stimulation experiments. Although the numbers of donors is relatively low in these experiments, the results suggested that combinations of streptococcal and *Veillonella* strains elicited an immune response profile that was distinct from the profile that was predicted on basis of the corresponding mono-stimulations. Some streptococci when combined with *Veillonella* substantially augmented IL-8, IL-6, IL-10, and TNF-α responses. Determining the exact mechanism underlying these co-stimulation effects is not trivial.

Our results imply that integrated responses to multiple bacteria or bacterial fragments could result in cytokine responses that are distinct from those anticipated on basis of the sum of single strain immune stimulation profiles. These observations may be particularly relevant for the mucosal-associated lymphoid tissue (e.g., Peyer's Patches' isolated lymphoid follicles), where multiple bacteria could be sampled by M-cells and transported to underlying dendritic cells. Cytokines induced by these DCs are predicted to influence activation of antigen-specific T cell responses after migration to the T cell areas of the PP or lymph nodes. Additionally, interaction bacteria or fragments of bacteria with CXCR1+ cells in the epithelium may influence local cytokine production and immunity. Our current knowledge and understanding of these interactions within the microbiota community as well as their interaction with the host (immune) system is an area of intense investigation but further mechanistic studies are needed to decipher the contribution of different bacteria and complex communities of bacteria to immunity and homeostasis. Immunomodulation analyses with a variety of well characterized bacterial isolates from the microbiota, would be a good starting point to identify potential immunomodulatory effects (including immunosuppression) for members of the microbiota. Deciphering of the underlying molecular mechanisms and identification of the bacterial effector molecules is a necessary subsequent step to unravel the molecular basis for individual bacteria-immune interactions. Insights in these individual molecular mechanisms of interaction for various bacterial species and strains could accelerate the deciphering of the complex and multifactorial interplay between the microbiota and the host immune system *in vivo*. In addition, high resolution *in vivo* measurements of the molecular responses to specific microbes can complement mechanistic *in vitro* studies by providing the necessary *in vivo* support for the molecular mechanisms unravelled with the help of *in vitro* systems. Mono-association (or simplified community colonization) studies in gnotobiotic animal models could provide an attractive reductionist model to extrapolate *in vitro* findings to an *in vivo* situation [55], [56], [57], [58], [59], [60]. Subsequent mono-association studies with derivatives of the same bacterial species or strains that lack one or more of their (immune) effector molecules could enable the *in vivo* establishment of the molecular interaction mechanisms proposed on basis of *in vitro* observations. As an example, approaches like this have elucidated how *B. fragilis* and its zwitterionic polysaccharide PSA are able to shape the host immune system (see [47], [48] for recent reviews). These reductionist *in vivo* and *in vitro* models offer a unique set-up to take the essential initial steps towards understanding the complexity of the interplay between the microbiota and the host in the intestine and its possible consequences for the overall physiology of the host organism, including its immune system status. Alternatively, the molecular responses elicited in the human intestine mucosa by specific bacteria can in some cases directly be determined *in vivo*, which is exemplified by the in depth analysis of transcriptional responses in the duodenal mucosa of healthy human volunteers upon the consumption of dietary lactobacilli [40], [45]. Such measurements may serve to guide *in vitro* studies that aim to decipher the underlying molecular mechanisms. The latter approach has the considerable advantage that the starting point for the *in vitro* mechanistic work is based on relevant *in vivo* observations in humans, and may therefore suffer less from the risk that responses in animal models cannot be translated to humans. In this context, it would be of interest to determine the *in vivo* (or *ex vivo*) small intestine mucosal responses to the typical small intestinal microbiota representatives that were studied here [17], [26], to generate reference datasets to guide *in vitro* mechanistic studies aimed to unravel the immunomodulatory capacities of these microbes.

## Supporting Information

Figure S1 **Histograms of the cytometry results.**
(EPS)

Table S1 **Statistical analysis of the MFI of stained cell surface markers CD83 (upper right panel) and CD86 (lower left panel) by monocyte derived dendritic cells stimulated at a cell to bacteria ratio of approximately 1∶1 and 1∶10.**
(DOCX)

Table S2 Average and SEM cytokine response values from monocyte derived iDCs* stimulated with bacterial strains.
(DOCX)

Table S3 **Statistical analysis of the cytokine responses (IL-8, upper right panel; IL-1β, lower left panel) by monocyte derived dendritic cells after stimulation with bacterial strains.**
(DOCX)

Table S4 **Statistical analysis of the cytokine responses (IL-6, upper right panel; IL-10, lower left panel) by monocyte derived dendritic cells after stimulation with bacterial strains.**
(DOCX)

Table S5 **Statistical analysis of the cytokine responses (TNF-α, upper right panel; IL-12p70, lower left panel) by monocyte derived dendritic cells after stimulation with bacterial strains**.
(DOCX)

Table S6 Average and SEM cytokine response values from monocyte derived iDCs* stimulated with bacterial strains with and without ***V. parvula*** **co-stimulation.**
(DOCX)
