# Supplementary material for: Immunomodulatory Properties of Streptococcus and Veillonella Isolates from the Human Small Intestine Microbiota
Source: PLoS One. 2014 Dec 5;9(12):e114277. doi: 10.1371/journal.pone.0114277 (PMC4257559; doi:10.1371/journal.pone.0114277)
Supplement: Table S1 — Statistical analysis of the MFI of stained cell surface markers CD83 (upper right panel) and CD86 (lower left panel) by monocyte derived dendritic cells stimulated at a cell to bacteria ratio of approximately 1∶1 and 1∶10. (DOCX) [file pone.0114277.s002.docx]

Table S1: Statistical analysis of the MFI of stained cell surface markers CD83 (upper right panel) and CD86 (lower left panel) by monocyte derived dendritic cells stimulated at a cell to bacteria ratio of approximately 1:1 and 1:10.

| **CD83**  **CD86** | medium | *S. parasanguinis* 1:1 | *S. parasanguinis* 1:10 | *S. equinus* 1:1 | *S. equinus* 1:10 | *S. salivarius 1* 1:1 | *S. salivarius 1* 1:10 | *S. salivarius 2* 1:1 | *S. salivarius 2* 1:10 | *S. salivarius 3* 1:1 | *S. salivarius 3* 1:10 | *S. salivarius 4* 1:1 | *S. salivarius 4* 1:10 | *V. parvula* 1:1 | *V. parvula* 1:10 | *E. gallinarum* 1:1 | *E. gallinarum* 1:10 |
| --- | --- | --- | --- | --- | --- | --- | --- | --- | --- | --- | --- | --- | --- | --- | --- | --- | --- |
| medium |  | 0.0570 | 0.0005 | 0.3238 | 0.0807 | 0.0185 | 0.0143 | 0.2186 | 0.0020 | 0.0859 | 0.0229 | 0.0216 | 0.0027 | 0.0214 | 0.0032 | 0.1412 | 0.0086 |
| *S. parasanguinis* 1:1 | 0.0046 |  | 0.0021 | 0.7884 | 0.3883 | 0.4787 | 0.0365 | 0.3721 | 0.0139 | 0.6004 | 0.0602 | 0.5254 | 0.0099 | 0.0806 | 0.0221 | 0.6417 | 0.0229 |
| *S. parasanguinis* 1:10 | 0.0003 | 0.1126 |  | 0.0272 | 0.2591 | 0.0022 | 0.2294 | 0.0011 | 0.4264 | 0.0555 | 0.3614 | 0.0025 | 0.2451 | 0.7979 | 0.5412 | 0.1259 | 0.1770 |
| *S. equinus* 1:1 | 0.1099 | 0.7320 | 0.1978 |  | 0.3845 | 0.5652 | 0.0406 | 0.8283 | 0.0715 | 0.5595 | 0.0648 | 0.5811 | 0.0193 | 0.0971 | 0.0754 | 0.5805 | 0.0276 |
| *S. equinus* 1:10 | 0.0282 | 0.6567 | 0.5698 | 0.5418 |  | 0.5212 | 0.1110 | 0.2237 | 0.4928 | 0.6780 | 0.1732 | 0.5137 | 0.0974 | 0.3342 | 0.4726 | 0.7445 | 0.0836 |
| *S. salivarius* 1 1:1 | 0.0021 | 0.8825 | 0.1131 | 0.6640 | 0.7092 |  | 0.0443 | 0.1240 | 0.0195 | 0.8406 | 0.0735 | 0.9562 | 0.0129 | 0.1059 | 0.0324 | 0.8304 | 0.0281 |
| *S. salivarius* 1 1:10 | 0.0041 | 0.0863 | 0.3366 | 0.1029 | 0.2456 | 0.0911 |  | 0.0259 | 0.1574 | 0.0626 | 0.8080 | 0.0440 | 0.6284 | 0.3727 | 0.1717 | 0.0768 | 0.9700 |
| *S. salivarius* 2 1:1 | 0.0364 | 0.5276 | 0.0605 | 0.9218 | 0.4085 | 0.4328 | 0.0541 |  | 0.0061 | 0.3081 | 0.0423 | 0.1440 | 0.0060 | 0.0493 | 0.0100 | 0.3874 | 0.0159 |
| *S. salivarius* 2 1:10 | 0.0006 | 0.5737 | 0.1680 | 0.5111 | 0.8697 | 0.6507 | 0.1165 | 0.2582 |  | 0.1600 | 0.2528 | 0.0206 | 0.1317 | 0.5387 | 0.9230 | 0.2710 | 0.1163 |
| *S. salivarius* 3 1:1 | 0.0053 | 0.6504 | 0.3233 | 0.5385 | 0.9033 | 0.7217 | 0.1518 | 0.3425 | 0.9656 |  | 0.1010 | 0.8252 | 0.0337 | 0.1715 | 0.1646 | 0.9597 | 0.0432 |
| *S. salivarius* 3 1:10 | 0.0105 | 0.1672 | 0.5225 | 0.1692 | 0.3660 | 0.1783 | 0.8435 | 0.1062 | 0.2235 | 0.2632 |  | 0.0729 | 0.8486 | 0.5288 | 0.2730 | 0.1212 | 0.7703 |
| *S. salivarius* 4 1:1 | 0.0009 | 0.7673 | 0.1129 | 0.6053 | 0.7585 | 0.8783 | 0.0958 | 0.3537 | 0.7371 | 0.7931 | 0.1889 |  | 0.0129 | 0.1049 | 0.0333 | 0.8185 | 0.0279 |
| *S. salivarius* 4 1:10 | 0.0055 | 0.1205 | 0.4502 | 0.1351 | 0.3161 | 0.1280 | 0.8615 | 0.0745 | 0.1644 | 0.2071 | 0.9728 | 0.1354 |  | 0.5395 | 0.1585 | 0.0552 | 0.5721 |
| *V. parvula* 1:1 | 0.0025 | 0.1204 | 0.5891 | 0.1525 | 0.3917 | 0.1275 | 0.6343 | 0.0697 | 0.1719 | 0.2389 | 0.8220 | 0.1346 | 0.7761 |  | 0.5828 | 0.2179 | 0.3236 |
| *V. parvula* 1:10 | 0.0001 | 0.0183 | 0.2129 | 0.0610 | 0.1988 | 0.0165 | 0.8092 | 0.0127 | 0.0208 | 0.0643 | 0.9804 | 0.0148 | 0.9846 | 0.7078 |  | 0.2652 | 0.1292 |
| *E. gallinarum* 1:1 | 0.0225 | 0.4247 | 0.9229 | 0.3745 | 0.7372 | 0.4571 | 0.4191 | 0.2655 | 0.5675 | 0.6162 | 0.5655 | 0.4879 | 0.5179 | 0.6527 | 0.4239 |  | 0.0558 |
| *E. gallinarum* 1:10 | 0.0006 | 0.0282 | 0.1811 | 0.0553 | 0.1579 | 0.0282 | 0.9381 | 0.0182 | 0.0366 | 0.0670 | 0.7684 | 0.0283 | 0.7805 | 0.5098 | 0.6732 | 0.3206 |  |

P-values ≤ 0.05 are highlighted in red
