# Supplementary material for: Immunomodulatory Properties of Streptococcus and Veillonella Isolates from the Human Small Intestine Microbiota
Source: PLoS One. 2014 Dec 5;9(12):e114277. doi: 10.1371/journal.pone.0114277 (PMC4257559; doi:10.1371/journal.pone.0114277)
Supplement: Table S2 — Average and SEM cytokine response values from monocyte derived iDCs* stimulated with bacterial strains. (DOCX) [file pone.0114277.s003.docx]

Table S2: Average and SEM cytokine response values from monocyte derived iDCs* stimulated with bacterial strains

| Bacterial strain | | IL-8 | | | IL-1β | | | IL-6 | | | IL-10 | | | TNF-α | | | IL-12p70 | | |
| --- | --- | --- | --- | --- | --- | --- | --- | --- | --- | --- | --- | --- | --- | --- | --- | --- | --- | --- | --- |
| *S. mitis* HSISM1 | | 8247 | ± | 1229 | 16 | ± | 3 | 1381 | ± | 634 | 29 | ± | 16 | 1457 | ± | 691 | 1416 | ± | 799 |
| *S. bovis* HSISB1 | | 5231 | ± | 1783 | 17 | ± | 4 | 161 | ± | 54 | 7 | ± | 2 | 86 | ± | 30 | 115 | ± | 53 |
| *S. salivarius* | HSISS1 | 16555 | ± | 2115 | 41 | ± | 5 | 2221 | ± | 403 | 58 | ± | 17 | 4732 | ± | 1220 | 1076 | ± | 358 |
|  | HSISS2 | 17147 | ± | 3018 | 41 | ± | 8 | 1104 | ± | 380 | 78 | ± | 30 | 2058 | ± | 811 | 556 | ± | 219 |
|  | HSISS3 | 10337 | ± | 1718 | 32 | ± | 4 | 696 | ± | 132 | 45 | ± | 10 | 2452 | ± | 783 | 81 | ± | 23 |
|  | HSISS4 | 11406 | ± | 2202 | 29 | ± | 3 | 1690 | ± | 400 | 46 | ± | 16 | 4933 | ± | 1134 | 1176 | ± | 539 |
| *V. parvula* HSIVP1 | | 13547 | ± | 4944 | 17 | ± | 4 | 3869 | ± | 1616 | 78 | ± | 40 | 1969 | ± | 1166 | 23 | ± | 14 |
| *E. gallinarum* HSIEG1 | | 17598 | ± | 2512 | 30 | ± | 8 | 4775 | ± | 2619 | 206 | ± | 147 | 5151 | ± | 2550 | 2397 | ± | 1109 |
| *L. plantarum* WCFS1** | | 27782 | ± | 1065 | 30 | ± | 3 | 4132 | ± | 85 | 128 | ± | 73 | 1229 | ± | 202 | 31 | ± | 9 |

*:Monocyte-derived iDCs were from five different healthy human donors

**: Cytokine responses determined using monocyte-derived DCs derived from 2 different human donors
