# Supplementary material for: Immunomodulatory Properties of Streptococcus and Veillonella Isolates from the Human Small Intestine Microbiota
Source: PLoS One. 2014 Dec 5;9(12):e114277. doi: 10.1371/journal.pone.0114277 (PMC4257559; doi:10.1371/journal.pone.0114277)
Supplement: Table S3 — Statistical analysis of the cytokine responses (IL-8, upper right panel; IL-1β, lower left panel) by monocyte derived dendritic cells after stimulation with bacterial strains. (DOCX) [file pone.0114277.s004.docx]

Table S3: Statistical analysis of the cytokine responses (IL-8, upper right panel; IL-1β, lower left panel) by monocyte derived dendritic cells after stimulation with bacterial strains.

| **IL-8**  **IL-1β** | *S. parasanguinis* | *S. equinus* | *S. salivarius 1* | *S. salivarius 2* | *S. salivarius 3* | *S. salivarius 4* | *V. parvula* | *E. gallinarum* |
| --- | --- | --- | --- | --- | --- | --- | --- | --- |
| *S. parasanguinis* |  | 0.1425 | 0.0188 | 0.0664 | 0.0151 | 0.0083 | 0.0589 | 0.0906 |
| *S. equinus* | 0.6179 |  | 0.0053 | 0.0083 | 0.0033 | 0.0019 | 0.0201 | 0.0185 |
| *S. salivarius 1* | 0.0009 | 0.0041 |  | 0.1395 | 0.6565 | 0.8334 | 0.9703 | 0.3055 |
| *S. salivarius 2* | 0.0267 | 0.0911 | 0.0949 |  | 0.1942 | 0.1127 | 0.2562 | 0.6791 |
| *S. salivarius 3* | 0.002 | 0.0104 | 0.4516 | 0.2675 |  | 0.7756 | 0.7498 | 0.467 |
| *S. salivarius 4* | 0.0007 | 0.0226 | 0.0322 | 0.9973 | 0.1274 |  | 0.8977 | 0.3254 |
| *V. parvula* | 0.9207 | 0.7125 | 0.0018 | 0.0432 | 0.0042 | 0.0048 |  | 0.4183 |
| *E. gallinarum* | 0.2438 | 0.7657 | 0.0018 | 0.0718 | 0.0046 | 0.0018 | 0.4057 |  |

P-values ≤ 0.05 are highlighted in red
