# Supplementary material for: Immunomodulatory Properties of Streptococcus and Veillonella Isolates from the Human Small Intestine Microbiota
Source: PLoS One. 2014 Dec 5;9(12):e114277. doi: 10.1371/journal.pone.0114277 (PMC4257559; doi:10.1371/journal.pone.0114277)
Supplement: Table S4 — Statistical analysis of the cytokine responses (IL-6, upper right panel; IL-10, lower left panel) by monocyte derived dendritic cells after stimulation with bacterial strains. (DOCX) [file pone.0114277.s005.docx]

Table S4: Statistical analysis of the cytokine responses (IL-6, upper right panel; IL-10, lower left panel) by monocyte derived dendritic cells after stimulation with bacterial strains.

| **IL-6**  **IL-10** | *S. parasanguinis* | *S. equinus* | *S. salivarius 1* | *S. salivarius 2* | *S. salivarius 3* | *S. salivarius 4* | *V. parvula* | *E. gallinarum* |
| --- | --- | --- | --- | --- | --- | --- | --- | --- |
| *S. parasanguinis* |  | 0.0915 | 0.2956 | 0.7182 | 0.321 | 0.6903 | 0.1897 | 0.2433 |
| *S. equinus* | 0.219 |  | 0.001 | 0.0396 | 0.0057 | 0.0053 | 0.051 | 0.1162 |
| *S. salivarius 1* | 0.2463 | 0.0166 |  | 0.0786 | 0.007 | 0.3771 | 0.3515 | 0.3635 |
| *S. salivarius 2* | 0.186 | 0.045 | 0.5762 |  | 0.3399 | 0.3193 | 0.1345 | 0.2029 |
| *S. salivarius 3* | 0.4206 | 0.0047 | 0.5123 | 0.3205 |  | 0.0458 | 0.0861 | 0.1584 |
| *S. salivarius 4* | 0.4647 | 0.0408 | 0.6276 | 0.3774 | 0.9366 |  | 0.227 | 0.2779 |
| *V. parvula* | 0.2802 | 0.1098 | 0.6472 | 0.9913 | 0.4329 | 0.4737 |  | 0.776 |
| *E. gallinarum* | 0.2636 | 0.2113 | 0.3447 | 0.4163 | 0.304 | 0.31 | 0.425 |  |

P-values ≤ 0.05 are highlighted in red
