# Supplementary material for: Immunomodulatory Properties of Streptococcus and Veillonella Isolates from the Human Small Intestine Microbiota
Source: PLoS One. 2014 Dec 5;9(12):e114277. doi: 10.1371/journal.pone.0114277 (PMC4257559; doi:10.1371/journal.pone.0114277)
Supplement: Table S5 — Statistical analysis of the cytokine responses (TNF-α, upper right panel; IL-12p70, lower left panel) by monocyte derived dendritic cells after stimulation with bacterial strains. (DOCX) [file pone.0114277.s006.docx]

Table S5: Statistical analysis of the cytokine responses (TNF-α, upper right panel; IL-12p70, lower left panel) by monocyte derived dendritic cells after stimulation with bacterial strains.

| **TNF-** α  **IL-12p70** | *S. parasanguinis* | *S. equinus* | *S. salivarius 1* | *S. salivarius 2* | *S. salivarius 3* | *S. salivarius 4* | *V. parvula* | *E. gallinarum* |
| --- | --- | --- | --- | --- | --- | --- | --- | --- |
| *S. parasanguinis* |  | 0.0826 | 0.0477 | 0.5879 | 0.3687 | 0.0307 | 0.7157 | 0.1996 |
| *S. equinus* | 0.143 |  | 0.0052 | 0.0411 | 0.0166 | 0.0027 | 0.1452 | 0.0823 |
| *S. salivarius 1* | 0.7083 | 0.0289 |  | 0.1054 | 0.1544 | 0.9067 | 0.1402 | 0.8858 |
| *S. salivarius 2* | 0.3298 | 0.0862 | 0.2501 |  | 0.736 | 0.0731 | 0.9512 | 0.2811 |
| *S. salivarius 3* | 0.1336 | 0.5704 | 0.0241 | 0.0633 |  | 0.1094 | 0.7398 | 0.3412 |
| *S. salivarius 4* | 0.8096 | 0.0856 | 0.8814 | 0.3175 | 0.0768 |  | 0.1058 | 0.9397 |
| *V. parvula* | 0.1196 | 0.1292 | 0.0187 | 0.0414 | 0.0627 | 0.0648 |  | 0.2893 |
| *E. gallinarum* | 0.4935 | 0.074 | 0.2901 | 0.1422 | 0.0703 | 0.3511 | 0.3511 |  |

P-values ≤ 0.05 are highlighted in red
