# Supplementary material for: Immunomodulatory Properties of Streptococcus and Veillonella Isolates from the Human Small Intestine Microbiota
Source: PLoS One. 2014 Dec 5;9(12):e114277. doi: 10.1371/journal.pone.0114277 (PMC4257559; doi:10.1371/journal.pone.0114277)
Supplement: Table S6 — Average and SEM cytokine response values from monocyte derived iDCs* stimulated with bacterial strains with and without V. parvula co-stimulation. (DOCX) [file pone.0114277.s007.docx]

Table S6: Average and SEM cytokine response values from monocyte derived iDCs* stimulated with bacterial strains with and without *V. parvula* co-stimulation

| Stimulation | Bacterial strain | | IL-8 | | | IL-1β | | | IL-6 | | | IL-10 | | | TNF-α | | | IL-12p70 | | |
| --- | --- | --- | --- | --- | --- | --- | --- | --- | --- | --- | --- | --- | --- | --- | --- | --- | --- | --- | --- | --- |
| Mono | *S. mitis* HSISM1 | | 9076 | ± | 3231 | 22 | ± | 0 | 301 | ± | 149 | 9 | ± | 2 | 197 | ± | 138 | 127 | ± | 64 |
|  | *S. bovis* HSISB1 | | 8377 | ± | 3648 | 24 | ± | 4 | 66 | ± | 38 | 9 | ± | 3 | 16 | ± | 3 | 8 | ± | 2 |
|  | *S. salivarius* | HSISS1 | 21125 | ± | 1561 | 33 | ± | 0 | 1597 | ± | 983 | 49 | ± | 16 | 2063 | ± | 576 | 239 | ± | 74 |
|  |  | HSISS2 | 23782 | ± | 3977 | 49 | ± | 14 | 280 | ± | 200 | 18 | ± | 1 | 151 | ± | 74 | 46 | ± | 12 |
|  |  | HSISS3 | 6933 | ± | 2972 | 24 | ± | 3 | 666 | ± | 414 | 41 | ± | 4 | 1208 | ± | 644 | 44 | ± | 14 |
|  |  | HSISS4 | 7571 | ± | 3399 | 24 | ± | 2 | 931 | ± | 322 | 33 | ± | 2 | 2613 | ± | 653 | 219 | ± | 113 |
|  | *V. parvula* HSIVP1 | | 6497 | ± | 3187 | 23 | ± | 2 | 1301 | ± | 97 | 26 | ± | 19 | 158 | ± | 91 | 4 | ± | 4 |
|  | *E. gallinarum* HSIEG1 | | 20916 | ± | 2055 | 47 | ± | 13 | 279 | ± | 119 | 18 | ± | 10 | 146 | ± | 77 | 248 | ± | 224 |
|  | *L. plantarum* WCFS1 | | 27782 | ± | 1065 | 30 | ± | 3 | 4132 | ± | 85 | 128 | ± | 73 | 1229 | ± | 202 | 31 | ± | 9 |
| Co | *S. mitis* HSISM1+ *V. parvula* HSIVP1 | | 22783 | ± | 1981 | 22 | ± | 1 | 3391 | ± | 260 | 62 | ± | 35 | 760 | ± | 139 | 73 | ± | 34 |
|  | *S. bovis* HSISB1+ *V. parvula* HSIVP1 | | 17253 | ± | 933 | 29 | ± | 1 | 1621 | ± | 121 | 33 | ± | 19 | 315 | ± | 183 | 10 | ± | 4 |
|  | *S. salivarius* + *V. parvula* HSIVP1 | HSISS1 | 24158 | ± | 606 | 38 | ± | 0 | 4604 | ± | 1537 | 135 | ± | 16 | 1391 | ± | 229 | 94 | ± | 36 |
|  |  | HSISS2 | 19222 | ± | 2100 | 28 | ± | 0 | 2637 | ± | 692 | 54 | ± | 0 | 838 | ± | 87 | 58 | ± | 22 |
|  |  | HSISS3 | 30054 | ± | 1937 | 55 | ± | 7 | 5451 | ± | 1446 | 154 | ± | 9 | 1516 | ± | 29 | 26 | ± | 1 |
|  |  | HSISS4 | 26390 | ± | 670 | 34 | ± | 0 | 4404 | ± | 1241 | 155 | ± | 50 | 1982 | ± | 347 | 98 | ± | 11 |
|  | *E. gallinarum* HSIEG1+ *V. parvula* HSIVP1 | | 26498 | ± | 2349 | 30 | ± | 1 | 4228 | ± | 1129 | 97 | ± | 26 | 1834 | ± | 35 | 300 | ± | 49 |
|  | *L. plantarum* WCFS1 + *V. parvula* HSIVP1 | | 4954 | ± | 1417 | 27 | ± | 4 | 35 | ± | 24 | 9 | ± | 6 | 5 | ± | 5 | 4 | ± | 4 |

*: iDCs were obtained from two different healthy human donors
